# Political Ideology and the Support or Opposition to United States Tobacco Control Policies

**DOI:** 10.1001/jamanetworkopen.2021.25385

**Published:** 2021-09-15

**Authors:** Sahil S. Shete, Robert Yu, Sanjay Shete

**Affiliations:** 1 Department of Clinical Psychology, University of Texas at Tyler, Tyler; 2 Department of Biostatistics, University of Texas MD Anderson Cancer Center, Houston; 3 Department of Epidemiology, University of Texas MD Anderson Cancer Center, Houston; 4 Division of Cancer Prevention and Population Science, University of Texas MD Anderson Cancer Center, Houston

## Abstract

This cross-sectional study examines the association between US adults’ self-reported political ideology and support of or opposition to policies that limit visibility and restrict advertisement of tobacco products.

## Introduction

Recently, political attitudes toward health care policies in the US have grown more divided between liberals and conservatives.^1^ Such political division has affected the public’s attitudes and acceptance of health initiatives,^2^ which the tobacco industry could potentially exploit in its efforts to weaken tobacco control policies. Healthy People 2020^3^ provides a framework for tobacco control, with initiatives such as limiting access to tobacco products, reducing tobacco advertising, and implementing hard-hitting antitobacco media campaigns. This cross-sectional study examined the associations between political ideology and support or opposition for various smoking policies. Such knowledge would allow us to develop interventions for a broader support of tobacco control.

## Methods

We obtained data from cycle 4 of the 2020 Health Information National Trends Survey (HINTS), a nationally representative survey of US adults.^4^ Data collection was conducted from February 24 to June 15, 2020. This study followed the Strengthening the Reporting of Observational Studies in Epidemiology (STROBE) reporting guideline for cross-sectional studies. HINTS data is exempt from institutional review board approval and informed consent in accordance with the Common Rule and University of Texas MD Anderson Cancer Center policy because the data are deidentified and publicly available.

Outcomes of interest were the support or opposition for various tobacco control policies, including: (1) requiring stores to keep tobacco products out of the customer’s view, (2) requiring stores to keep tobacco advertisements away from their registers and windows, (3) prohibiting tobacco products from being advertised on social media, (4) requiring warning labels with both images and words on cigarette packs, and (5) requiring that movies with smoking carry an R rating.

A multinomial logistic regression analysis was used to identify how political ideology was associated with the odds of support or opposition to tobacco control policies. The explanatory variable, political ideology, was assessed with the survey question, “Thinking about politics these days, how would you describe your own political viewpoint?” The analysis was adjusted for age, sex, self-reported race and ethnicity, smoking status, education level, household annual income, and urban/rural residency. The statistical significance level was defined as 2-sided *P* < .05. Statistical analyses were performed using survey analysis procedures in SAS/STAT software version 9.4 (SAS Institute).

## Results

The study sample included 3765 adults: 2204 women (weighted percentage, 51.4%), 2133 non-Hispanic White individuals (63.3%), and 436 (13.8%) current and 935 (23.0%) and former smokers. Furthermore, 828 (21.7%) self-reported as very conservative or conservative, 395 (11.8%) as somewhat conservative, 1200 (37%) as moderate, 368 (11.4%) as somewhat liberal, and 690 (18.2%) as very liberal or liberal. Compared with moderates, very liberal and liberal participants were more likely to say they strongly support or support requirements for stores to keep tobacco products out of the customer’s view (OR, 1.60; 95% CI, 1.11-2.32) and somewhat conservative participants were more likely to strongly oppose or oppose such requirements (OR, 2.33; 95% CI, 1.15-4.72) (Table 1). Likewise, compared with moderates, very liberal and liberal participants were more likely to strongly support or support requirements that stores keep tobacco advertisements away from registers and windows (OR, 1.70; 95% CI, 1.17-2.46) and somewhat conservative participants were more likely to strongly oppose or oppose such measures (OR, 2.76; 95% CI, 1.32-5.77).

**Table 1.** Attitudes Toward Preventive Measures Related to All Tobacco Products by Political Viewpoint

Political viewpoint^a^	Odds of supporting preventive measure, OR (95% CI)^b^		
	Requiring stores to keep tobacco products out of customer’s view	Requiring stores to keep tobacco advertisements away from their registers and windows	Tobacco products not being advertised on social media
Very conservative/conservative vs moderate			
Strongly support/support	1.02 (0.71-1.46)	1.08 (0.77-1.53)	1.06 (0.72-1.57)
Strongly oppose/oppose	1.06 (0.55-2.02)	1.45 (0.77-2.73)	1.33 (0.68-2.62)
Somewhat conservative vs moderate			
Strongly support/support	1.13 (0.71-1.78)	1.25 (0.84-1.86)	1.11 (0.70-1.75)
Strongly oppose/oppose	2.33 (1.15-4.72)	2.76 (1.32-5.77)	2.41 (0.99-5.88)
Somewhat liberal vs moderate			
Strongly support/support	1.54 (0.88-2.69)	1.68 (0.99-2.83)	1.79 (0.92-3.50)
Strongly oppose/oppose	1.40 (0.67-2.94)	1.27 (0.60-2.70)	1.22 (0.46-3.24)
Very liberal/liberal vs moderate			
Strongly support/support	1.60 (1.11-2.32)	1.70 (1.17-2.46)	1.98 (1.36-2.88)
Strongly oppose/oppose	0.99 (0.55-1.78)	1.08 (0.50-2.35)	1.53 (0.64-3.66)

Abbreviation: OR, odds ratio.

^a^ Participants were asked to identify their ideological beliefs with the following prompt: “Thinking about politics these days, how would you describe your own political viewpoint?” Response options included very liberal, liberal, somewhat liberal, moderate, somewhat conservative, conservative, and very conservative. Very liberal and liberal were collapsed into a single response category, and very conservative and conservative were also collapsed into a single response category. Moderate was used as the reference category.

^b^ Participants were asked to assess their attitudes towards tobacco-related policies with the following prompt: “To what extent would you support or oppose the following measures related to all tobacco products, including cigarettes, e-cigarettes, smokeless tobacco, hookah, and cigars?” Response options included strongly oppose, oppose, neither support nor oppose, support, and strongly support. Strongly oppose and oppose were collapsed into a single response category, and strongly support and support were also collapsed into a single response category. Response neither support nor oppose was used as the reference category for each of the outcome variables in the multinomial logistic regression. ORs adjusted for age, sex, race/ethnicity, education level, income, smoking status, and rural/urban residency.

Compared with moderates, very liberal and liberal participants were more likely to strongly support or support tobacco products not being advertised on social media (OR, 1.98; 95% CI, 1.36-2.88). This same group was also more likely to strongly support or support warning labels with both images and words on cigarette packs (OR, 1.68; 95% CI, 1.07-2.62), but were strongly opposed or opposed labeling movies with smoking with an R rating (OR, 1.64; 95% CI, 1.02-2.63) (Table 2).

**Table 2.** Attitudes Toward Preventive Measures Related to Cigarettes by Political Viewpoints

Political viewpoint^a^	Support for preventive measure, OR (95% CI)^b^	
	Movies with cigarette smoking should be rated R to protect children and youth	Cigarette packs should have warning labels with both images and words to show the negative health effects of smoking
Very conservative/conservative vs moderate		
Strongly support/support	1.36 (0.87-2.14)	1.29 (0.85-1.93)
Strongly oppose/oppose	1.38 (0.89-2.15)	1.56 (0.81-3.00)
Somewhat conservative vs moderate		
Strongly support/support	1.20 (0.74-1.97)	1.09 (0.65-1.82)
Strongly oppose/oppose	1.72 (0.79-3.74)	1.77 (0.85-3.67)
Somewhat liberal vs moderate		
Strongly support/support	1.35 (0.72-2.53)	1.27 (0.79-2.03)
Strongly oppose/oppose	1.35 (0.67-2.73)	2.13 (0.84-5.39)
Very liberal/liberal vs moderate		
Strongly support/support	1.38 (0.94-2.01)	1.68 (1.07-2.62)
Strongly oppose/oppose	1.64 (1.02-2.63)	1.13 (0.53-2.41)

Abbreviation: OR, odds ratio.

^a^ Participants were asked to identify their ideological beliefs with the following prompt: “Thinking about politics these days, how would you describe your own political viewpoint?” Response options included very liberal, liberal, somewhat liberal, moderate, somewhat conservative, conservative, and very conservative. Very liberal and liberal were collapsed into a single response category, and very conservative and conservative were also collapsed into a single response category. Moderate was used as the reference category.

^b^ Participants were asked to assess their attitudes towards cigarette-related policies with the following prompt: “To what extent would you support or oppose the following measures related to cigarettes?” Response options included strongly oppose, oppose, neither support nor oppose, support, and strongly support. Strongly oppose and oppose were collapsed into a single response category, and strongly support and support were also collapsed into a single response category. Response neither support nor oppose was used as the reference category for each of the outcome variables in the multinomial logistic regression. ORs adjusted for age, sex, race/ethnicity, education level, income, smoking status, and rural/urban residency.

## Discussion

Our findings suggest that individuals identifying as liberals support removing tobacco products from customers’ view and keeping pro-tobacco advertisements away from registers and windows, while conservatives oppose these evidence-based tobacco control measures. Furthermore, liberals were more likely to support tobacco products not being advertised on social media and having warning labels with both images and words on cigarette packs; however, they opposed giving R ratings to movies that depict smoking.

These differing political viewpoints should be accounted for when educating the public against industry-sponsored pro-tobacco campaigns^5^ that currently exist in retail stores, packaging, movies, and social media. Furthermore, with the politicization of other public health policies increasing,^1^ it is important to keep raising awareness of the negative health effects tobacco has at the individual and population levels so these policies do not manifest into another partisan issue. We call for tailoring educational messages about the harms of tobacco to political subgroups that appeal to their societal values, as similar strategies have been shown to be effective with other health policies (eg, childhood obesity prevention). Tobacco advertisements have been especially potent for underserved populations^6^ (eg, racial/ethnic minority populations); therefore, preventing political ideology from becoming another vulnerability is critical as well. Study limitations included a low response rate and a cross-sectional design, which could not establish causal relationships.
